# Radiotherapy of granulomatosis with polyangiitis occurring in the eyelid: A case report and literature review

**DOI:** 10.1097/MD.0000000000022794

**Published:** 2021-01-22

**Authors:** Jinlong Wei, Qin Zhao, Min Yao, Lingbin Meng, Ying Xin, Xin Jiang

**Affiliations:** a Department of Radiation Oncology; b Jilin Provincial Key Laboratory of Radiation Oncology & Therapy, The First Hospital of Jilin University; c NHC Key Laboratory of Radiobiology, School of Public Health, Jilin University; d Department of pathology, the Second Hospital of Jilin University, Changchun, China; e Department of Internal Medicine, Florida Hospital, Orlando, FL 32803, USA; f Key Laboratory of Pathobiology, Ministry of Education, Jilin University, Changchun, China.

**Keywords:** case report, eyelid, granulomatosis with polyangiitis, radiotherapy, treatment

## Abstract

**Introduction:**

Granulomatosis with polyangiitis (GPA) is a chronic systemic vasculitis characterized by necrotizing granulomatous vasculitis. The disease mainly affects the middle and small blood vessels and mainly occurs in the upper respiratory tract (nose and paranasal sinuses), lower respiratory tract (lungs), and kidneys. Disease occurrence in the eyelid area is relatively rare. The standard GPA treatment is combination therapy with adrenocortical hormone and immunosuppressants. Radiotherapy as a treatment option for GPA has not been widely investigated.

**Patient concerns:**

A 29-year-old man presented with a 1.0 × 1.0 cm mass without exophthalmos and decreased vision in the left lower eyelid. Computed tomography revealed a mass-like high-density shadow below the left eye with a computed tomography value of 80-108 U.

**Diagnosis:**

The laboratory investigations revealed positive cytoplasmic antineutrophil cytoplasmic antibodies (titer = 1:40). Biopsy of the lower left eyelid mass revealed necrosis and granulomatous reaction with a large number of inflammatory cell infiltration. After consultation with the pathology department, the diagnosis was determined as left lower eyelid GPA.

**Interventions:**

The patient received 9MeV electron beam radiation therapy in the area of the left lower eyelid lesion.

**Outcomes:**

The lesion in the patient was significantly reduced and the symptom relieved obviously. No symptom recurrence or significant toxicity occurred during or after the treatment. The patient remains under routine follow-up.

**Conclusion:**

We present a case of a male patient with GPA located exclusively in the eyelid area, who underwent successful radiotherapy and achieved a complete response. The lesson we learned from this case study is that for GPA patients, when the standard treatment model fails to achieve good results, novel treatments such as radiotherapy should be considered according to the situation.

## 1 Introduction

Granulomatosis with polyangiitis (GPA), previously known as Wegener Granulomatosis, is a chronic systemic vasculitis. The lesions mainly affect small and medium blood vessels and mainly occur respiratory tract and kidneys. GPA is a rare disease with an incidence of 3 per 100,000 people.^[1]^ The diagnosis of GPA is based on clinical and histopathological examinations. According to the criteria of the American College of Rheumatology (1990), GPA is defined by the presence of at least 2 of the following 4 criteria:

(1) sinus involvement;
(2) lung radiograph showing nodules, a fixed pulmonary infiltrate or cavities;
(3) urinary sediment with hematuria or red cell casts;
(4) histological granulomas within an artery or in the perivascular area of an artery or arteriole. ^[2]^

Laboratory tests play an essential role in the diagnosis of GPA. Elevated cytoplasmic antineutrophil cytoplasmic antibodies (c-ANCA) level is a helpful diagnostic marker for GPA.^[3]^

The standard GPA treatment is combination therapy with adrenocortical hormone and immunosuppressants. Radiotherapy may be used in select GPA patients, who are refractory to standard treatment.^[4]^ Only a few GPA cases treated with radiotherapy have been published in the literature, but there is no report on the effects of radiotherapy on GPA eyelid lesions. A case report of a male patient with GPA exclusively located in the eyelid area, who underwent radiotherapy and had a complete response, is hereby presented.

## 2 Case presentation

**Figure 1:**
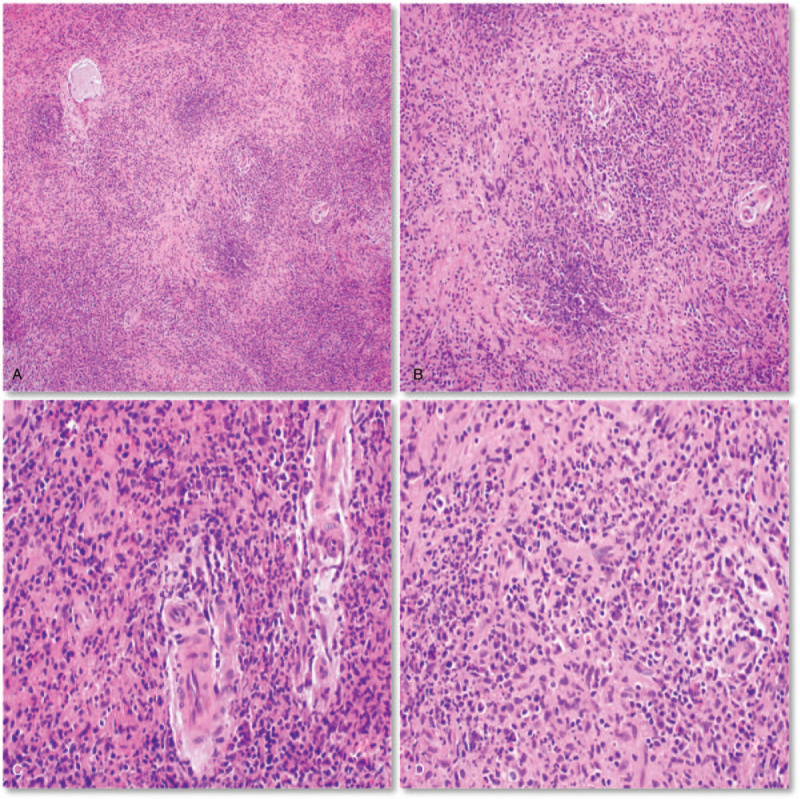
Histopathology of granulomatosis with polyangiitis (GPA). A: A micrograph of lesion in the eyelid area biopsy depicted necrosis and granulomatous reaction with a large number of inflammatory cells infiltration (H&E staining, original magnifications × 100); B: Infiltration of histiocytes and multicellular giant cells was observed around the necrosis without typical granulomatous inflammatory infiltrate (H&E staining, original magnifications × 200); C: The wall of a part of a blood vessel in the center was damaged and neutrophil infiltration was mainly around (H&E staining, original magnifications × 400); D: The infiltrated cells in the background were neutrophils, lymphocytes, plasma cells and eosinophils. Most of them were neutrophil infiltration (H&E staining, original magnifications × 400).

A 29-year-old man presented with a 1.0 × 1.0 cm mass without exophthalmos and decreased vision in the left lower eyelid, in March 2017. The patient had no history of trauma or foreign body/infection. The laboratory investigations revealed positive c-ANCA (titer = 1:40). Biopsy of the lower left eyelid mass was performed in March 2017, which revealed necrosis and granulomatous reaction with a large number of inflammatory cell infiltration (Fig. 1A-D). After consultation with the pathology department, the diagnosis was determined as left lower eyelid GPA. Other parts of the patient were assessed and no abnormalities were found. The patient had been treated 40 mg daily methylprednisolone and 0.2 g daily cyclophosphamide since March 2017, without the lesions being subsided. The mass progressively increased to 3.0 × 2.0 cm in the past 6 months. Computed tomography (CT) was performed in August 2017, which revealed a mass-like high-density shadow below the left eye with a CT value of 80 to 108 U.

**Figure 2:**
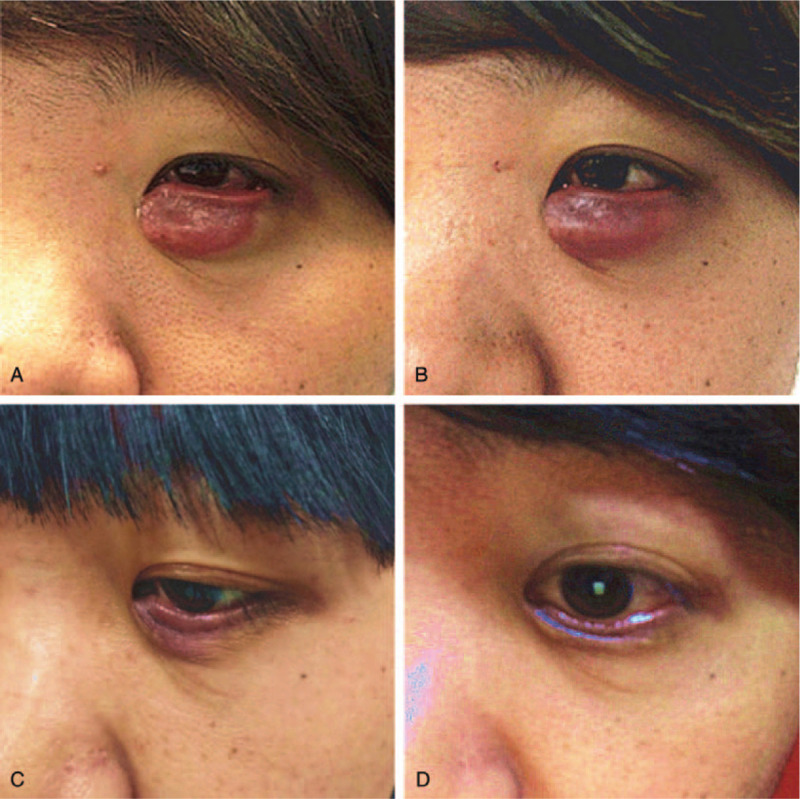
The photograph of the eye at different time. A: The eye before radiotherapy; B: The eye at 3 days after radiotherapy; C: The eye at 5 months after radiotherapy; D: The eye at 1 year after radiotherapy.

**Figure 3:**
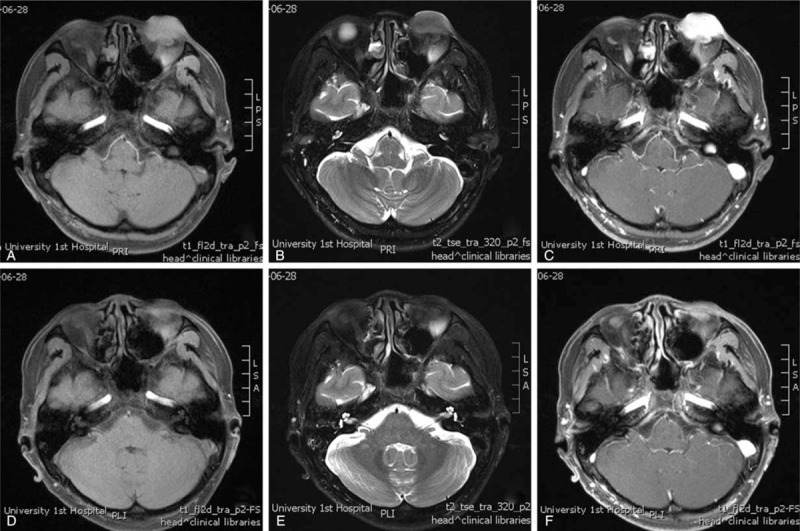
Magnetic resonance imaging (MRI) of the eye. A, B and C: The MRI of the eye before radiotherapy showed an abnormal signal of lumps in the lower left eyelid on T1 and T2 imaging; D, E and F: The MRI of the eye at 5 months after radiotherapy showed a slightly abnormal signal in the lower left eyelid on T1 and T2 imaging.

Photography of the eye before radiotherapy revealed a 3.0 × 2.0 cm red mass in the left lower eyelid (Fig. 2. A). Magnetic resonance imaging (MRI) of the eye before radiotherapy revealed an abnormal lump in the lower left eyelid and the lower front of the left eye with an approximate size of 1.4 × 2.1 × 3.2 cm. Enhanced scans were clearly and uniformly enhanced. The lesion partially surrounded the left eyeball (Fig. 3. A, B and C).

Radiotherapy in localized lesions was planned and performed in September 2017, following discussions with the multiple disciplinary team. Under the Varian linear accelerator, 9MeV electron beam radiation therapy was applied to the area of the left lower eyelid lesion. The irradiation field was defined as a 5mm areal expansion of the left lower eyelid lesion. The totaling dose was 30 Gy/2.0 Gy/15 F. The patient developed no adverse reactions to radiotherapy.

Routine follow-up was performed since the end of treatment. Photography of the eye 3 days after radiotherapy revealed that no change in the size of the tumor was observed (Fig. 2. B). But, we found that the mass was significantly softer than its previous texture. The tumor progressively reduced 1 month after radiotherapy. He returned to the hospital 5 months after radiotherapy and eye photography revealed no obvious mass in the lower left eyelid and the eyelid looked slightly raised and dark red (Fig. 2. C). The MRI revealed a slightly thicker left lower eyelid. Abnormal signals below the left eyeball also disappeared. The left lower orbital mass significantly improved compared to the MRI before radiotherapy (Fig. 3. D, E, and F).

The patient returned to the hospital 8 months after radiotherapy and eye photography revealed no obvious mass in the lower left eyelid. The eyelid appeared slightly raised, dark red, and soft (Fig. 2. D).

## 3 Discussion

GPA is a chronic systemic vasculitis and a rare disease with an incidence of 3 per 100,000 people. More than half of GPA cases occur between the ages of 30 and 50, as GPA is rare among children and teenagers. The annual incidence of childhood and teenage GPA is 1-2 per 1,000,000 people.

Clinical manifestations of GPA in regards to different parts of the lesion and varying degrees, include cough, hemoptysis, fever, night sweats, asthma, dyspnea, nasal discharge, nasal mucosal ulceration, hearing loss, altered voice, skin rash, joint swelling and hematuria.^[5]^ The clinical manifestations of orbital GPA include exophthalmos, decreased vision, scleritis, eye pain and eye infections.^[6]^ Severe clinical manifestations may result in complete loss of vision and permanent facial deformity.^[3]^ Nevertheless, the clinical manifestations of GPA located in the eyelid is rarely reported.

Currently, laboratory tests play an essential role in diagnosis of GPA. Serological indicators of inflammation, including erythrocyte sedimentation rate and C-reactive protein levels, are frequently elevated in this disease, whereas C3 and C4 complement levels may be reduced.^[3,7]^ Elevated c-ANCA levels are a highly helpful diagnostic tool for GPA.^[8]^ In a meta-analysis, positive c-ANCA serology was 91% sensitive and 99% specific in GPA patients.^[9]^ In our case, laboratory investigations revealed positive c-ANCA.

Before immunomodulatory therapy was introduced in the 1970 s, GPA had a poor prognosis. The median survival time for GPA patients was 5 months. The mortality rate of GPA was higher than 80% within 1 year after diagnosis.^[10]^ Since 1970, synthetic glucocorticoid combined with immunosuppressant combination therapy has become the gold standard for GPA treatment with a significantly improved therapeutic effect on patients. With this regimen, the 5-year survival rate is 95% and the 10-year survival rate 80%.^[11]^ Cyclophosphamide was the first choice for the treatment of granulomatous lesions, which can be used for at least 1 year. Prednisone significantly reduces inflammatory changes in blood vessels.^[12]^ Although immunosuppressive therapy can prolong survival, it can also lead to severe adverse reactions.^[10]^

Rituximab therapy has recently become an attractive new therapy for GPA. Some clinical studies have reported that the rituximab is effective in treating orbital GPA.^[13]^ At present, Rituximab has been approved for GPA by the FDA. Studies have shown that infliximab (chimeric human-mouse monoclonal antibody IgG1, combined with tumor necrosis factor alpha) also has a good therapeutic effect against GPA, but it is still under clinical investigation.^[14]^ Additional operative treatment may contribute to decompression in certain GPA cases that present with severe pain or exophthalmos.^[15]^

Resistance to standard systemic treatment could present a significant modern-age problem. Radiotherapy may be used in selected GPA patients, who are refractory to standard treatment. Data on radiotherapy outcomes in the literature are limited to date so far, but case reports show significant response rates.^[4,16]^

There are several GPA cases that acquire a good curative effect from radiotherapy. In an isolated case report of a female patient with GPA located in the facial area, the patient received complete radiotherapy and achieved complete remission. The patient's lesion was located at the base of the nose near the right inner corner of the eye. In this case, the patient was treated with radiotherapy after failure of the combination therapy with cortical hormone and immunosuppressants. Intensity-modulated radiation therapy was administered to local facial lesions and the radiation dose was 30 Gy/2.0 Gy/15 F. The follow-up time was 3 years. During the period of regular inspection, the appearance changes and CT scans indicated that the lesions continued to shrink and there was no symptom recurrence.^[17]^

Despite the small number of studies on radiotherapy for GPA they all reported good efficacy with low side effects, indicating that radiotherapy has broad prospects as an advanced GPA treatment model. Whether radiotherapy can become a routine treatment for GPA is still unclear and in need of further clinical investigation.

## Acknowledgments

We would like to thank Editage (www.editage.cn) for English language editing.

## Author contributions

JX and YX conceived and designed the study. YM wrote and reviewed the pathological images and labels. WJL and ZQ wrote the paper. MLB and XY reviewed and edited the manuscript. All authors read and approved the manuscript.

**Funding acquisition:** Ying Xin.

**Visualization:** Min Yao.

**Writing – original draft:** Jinlong Wei, Qin Zhao.

**Writing – review & editing:** Lingbin Meng, Ying Xin.
